# Unraveling the Origins and Drivers of Potentially Toxic Elements (PTEs): A Sequential Framework Integrating Receptor Model and Machine Learning

**DOI:** 10.3390/toxics14060525

**Published:** 2026-06-17

**Authors:** Jingyun Wang, Xiaofeng Zhao, Jiufen Liu, Yunxian Yan, Wei Zhao, Chuanbo Xia, Jianye Zheng, Jiwei Liu

**Affiliations:** 1 Key Laboratory of Coupling Process and Effect of Natural Resources Elements, Beijing 100055, China; 2 Key Laboratory of Gold Mineralization Processes and Resource Utilization, MNR, Key Laboratory of Metallogenic Geological Process and Resource Utilization of Shandong Provincial, Shandong Institute of Geological Sciences, Jinan 250013, China; 3 Institute of Geographic Sciences and Natural Resources Research, Chinese Academy of Sciences, Beijing 100101, China

**Keywords:** cadmium, source apportionment, driving mechanisms, APCS-MLR, machine learning models

## Abstract

Source apportionment and the elucidation of driving mechanisms are essential for targeted soil pollution management. This study investigated surface soils across six towns in southern Shimen County, northwestern Hunan Province, where 662 samples were collected to determine the concentrations of As, Cd, Cr, Cu, Ni, Pb, and Zn. Multivariate statistics and the APCS-MLR receptor model were integrated to quantify pollution sources, while three machine learning models (RF, XGBoost, and LightGBM) were applied to identify key drivers of the spatial enrichment of Cd. Results showed that Cd was significantly enriched, with a mean concentration of 0.43 mg/kg (3.41 times the provincial background value). The mean concentrations of As, Cr, Cu, Ni, Pb and Zn were 11.97 mg/kg, 81.01 mg/kg, 24.15 mg/kg, 49.25 mg/kg, 29.56 mg/kg and 76.77 mg/kg, respectively, and these PTEs remained at normal background levels. Significant inter-element correlations indicated common sources. Three primary sources were quantified—natural parent material (43.83%), mining activities (30.99%), and mixed sources of coal mining and agricultural inputs (7.84%), with 17.34% attributed to unidentified mixed sources. Natural sources dominated the geogenic enrichment of Cd, Cu, Ni, Pb, and Zn; mining activities governed the accumulation of As, Cr, Cu, and Pb; a mixed source of coal mining and agricultural practices contributed substantially to Cd enrichment. Machine learning identified PM10, topography, strata, and soil type as dominant drivers, with their total feature importance reaching 70.05%. Among these factors, natural factors and anthropogenic factors accounted for 44.23% and 55.77% of the total feature importance, in turn revealing coupled natural–anthropogenic controls. This study establishes an integrated framework linking source apportionment and driver identification, providing scientific insights for potentially toxic elements (PTEs) control in analogous mining–agricultural regions.

## 1. Introduction

With the rapid development of the economy and the continuous advancement of industrialization and agricultural intensification, the issue of soil PTE pollution has become increasingly severe, emerging as a global ecological and environmental concern [1,2,3]. Soil PTEs are characterized by their persistence, tendency to accumulate, and strong concealment, and they can enter the human body through pathways such as the food chain, posing significant risks to human health [4,5]. Hunan Province, known as China’s “hometown of non-ferrous metals,” experiences intensive human activities such as industrial and mining operations and agricultural production, making it a key region for PTE accumulation and pollution control [6,7]. Against this background, accurately identifying the sources of regional soil PTE pollution and elucidating their driving mechanisms holds important theoretical value for fundamental research on soil PTE pollution and provides practical guidance for developing targeted pollution control and remediation strategies.

Source apportionment and causal identification of PTE pollution serve as important prerequisites for regional environmental risk management. The sources of PTEs in soil are complex, influenced by both natural factors and anthropogenic activities [8]. Multivariate statistical analysis, with its strengths in dimensionality reduction, information condensation, and clustering, enables the extraction of latent pollution patterns and key variation features from multidimensional environmental datasets. By mitigating analytical biases introduced by multicollinearity, it is a well-established method for deconstructing pollution characteristics and identifying source factors [9,10]. Receptor models do not rely on detailed emission source inventories but instead use measured environmental receptor data to identify pollution source types and quantitatively apportion source contributions. They have been widely applied to the source apportionment of PTEs in soil, sediments, and atmospheric particulates [11,12]. Among these, the APCS-MLR model integrates the technical advantages of principal component analysis and multiple linear regression. By using absolute principal component scores to effectively correct the issue of negative factor scores, it can both qualitatively identify the types of potential pollution sources in the study area and quantitatively estimate the contribution shares of various sources. The model has clear physical meaning and strong interpretability, making it suitable for source tracing and quantitative contribution analysis of regional PTE pollution [13,14].

The spatial enrichment and differentiation of PTEs in soil are governed by the interplay of natural background, geographical conditions, and anthropogenic activities, forming complex nonlinear relationships with environmental factors and soil physicochemical properties [15,16]. While receptor models can quantify potential pollution sources, they often fail to capture underlying mechanisms and objectively assess the relative contributions of specific drivers. Machine learning, with its robust nonlinear fitting capability and nonparametric flexibility, has emerged as a powerful tool for disentangling these complex relationships and has become the prevailing method for identifying dominant factors controlling PTE dynamics [17,18].

Accordingly, this study focuses on PTEs in southern Shimen County, Hunan Province. It is hypothesized that PTE distribution is jointly controlled by multiple pollution sources and environmental factors. Multivariate statistical analysis and the APCS-MLR receptor model were first employed to qualitatively identify and quantitatively apportion potential pollution sources. Furthermore, we aim to clarify the key factors dominating spatial variations of PTEs, so three integrated tree models—Random Forest, XGBoost, and LightGBM—were adopted to systematically analyze the nonlinear driving effects and dominant contribution characteristics of environmental factors on the spatial distribution of PTEs in soil. The integrated framework coupling pollution source apportionment and driving mechanism analysis can provide a reference research paradigm for tracing PTE sources and exploring accumulation mechanisms in complex natural–anthropogenic affected areas. Meanwhile, it offers scientific data support and a theoretical basis for targeted prevention and control, ecological risk management, and comprehensive remediation of regional soil pollution.

## 2. Materials and Methods

### 2.1. Study Area, Sampling, and Chemical Analysis

**Figure 1:**
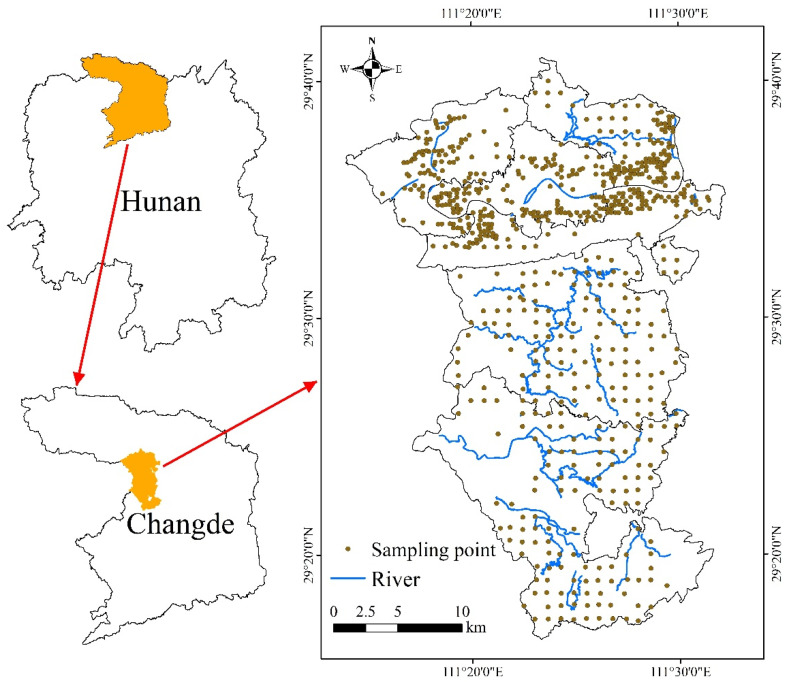
Spatial distribution of sampling points.

The study area covers six townships in southern Shimen County, Hunan Province, China. It falls within the transitional monsoon climate zone between the mid-subtropical and typical subtropical regions, with a mean annual temperature of 18.4 °C and an average annual precipitation of 1390.3 mm. Field sampling was conducted in 2015, yielding a total of 662 surficial samples (0–20 cm) (Figure 1). The geographic coordinates of each site were recorded using a portable GPS device. All collected soil samples were sealed in plastic bags and promptly transported to the laboratory for subsequent pretreatment and analysis. Prior to testing, the soil samples were air-dried at ambient temperature, ground into fine particles, and sieved through a 200-mesh nylon sieve with a pore size of 0.149 mm. Approximately 0.2 g of soil was weighed and digested with HNO_3_–H_2_O_2_ mixture following USEPA Method 3050B [19]. All reagents were guaranteed reagent (GR) grade. The resultant digest was cooled, filtered, and adjusted to 50 mL with ultrapure water before being analyzed by instruments. Arsenic (As) was determined using atomic fluorescence spectrometry (Beijing Kechuang Haiguang Instrument Co., Ltd., Beijing, China). Cadmium (Cd), chromium (Cr), copper (Cu), nickel (Ni), lead (Pb), and zinc (Zn) were measured using inductively coupled plasma mass spectrometry (ELAN DRC-e ICP-MS, PerkinElmer, Waltham, MA, USA). National standard reference materials (GSS-1, GSS-4) were used for analytical quality control throughout the experiment. In addition, blank samples and duplicate samples (one duplicate per 20 samples) were routinely arranged. The standard reference materials and field samples adopted the same pretreatment and determination procedures. The recoveries of the certified reference materials GSS-1 and GSS-4 were in the range of 90–110%, meeting the requirements for analytical quality control.

### 2.2. Influencing Factors

PTEs in Soil are affected by the combined effects of natural and anthropogenic factors [20]. In this study, 10 potential driving factors influencing PTEs in soil were selected for analysis. Natural factors include strata, soil type, topography, and slope; anthropogenic factors include land use type, GDP, population density, atmospheric deposition, distance to roads, and distance to rivers. Notably, soil types in this study were classified based on the Genetic Soil Classification of China (GSCC), the official system used for the Second National Soil Survey of China [21]. The data sources and spatial distribution of these driving factors are shown in Table S1 and Figure S1.

Due to data availability, this study collected the main driving factors affecting PTEs in soil as comprehensively as possible, all of which have been proven to exert significant influences on PTE concentrations. Differences in the weathering of strata and lithology determine the initial background concentrations of PTEs in soil and form the fundamental natural basis for their spatial distribution. Differences in physicochemical properties among various soil types directly affect the migration and transformation processes of PTEs and alter their enrichment and differentiation characteristics. Topography, landforms and slope regulate surface material migration and slope confluence processes, thereby reshaping the spatial distribution patterns of PTEs. Land use type reflects the differences in anthropogenic disturbance and agricultural input intensity, and directly affects the exogenous accumulation of PTEs. GDP and population density represent the intensity of regional economic development and human activity disturbance, respectively; higher values indicate more pronounced superimposed impacts of anthropogenic pollution. Atmospheric dust deposition is an important exogenous input pathway for PTEs, and continuous deposition aggravates PTE enrichment in soil. Meanwhile, traffic pollutant emissions and river irrigation inputs also play critical driving roles in the accumulation and spatial differentiation of PTEs.

### 2.3. Methods

In this study, multivariate statistical analysis was used to identify potential sources of PTEs, and the APCA-MLR model was applied to quantify the contribution rate of each source. Meanwhile, three widely used machine learning algorithms were adopted to determine the key driving factors of Cd in the study area. The detailed methods are described as follows:

#### 2.3.1. Multivariate Statistical Analysis

Correlation analysis and principal component analysis (PCA) are two of the most classical and widely used methods in multivariate statistical analysis. They can effectively explore the intrinsic relationships among elements and identify pollution source characteristics, and have therefore been extensively applied to the source apportionment of PTEs in soil [22,23]. Correlation analysis can quantitatively characterize the correlation degree between PTEs. Generally, a larger absolute value of the correlation coefficient indicates a more significant linear correlation, implying similar geochemical behaviors and a higher probability of being controlled by the same geological genesis or anthropogenic activities, which provides a reliable basis for preliminary discrimination of the homology of PTEs [24]. PCA realizes dimensionality reduction and information compression of multidimensional data. On the premise of retaining the maximum original information, it transforms numerous highly correlated PTEs into a small number of independent comprehensive principal components. According to the loading characteristics of each element on the principal components, potential pollution sources such as natural parent material, mining activities, agricultural fertilization and pesticide application can be objectively distinguished. Hence, PCA is an important tool for the qualitative source identification of PTEs in soil [24].

#### 2.3.2. Absolute Principal Component Score-Multiple Linear Regression (APCS-MLR)

APCS-MLR is a receptor model combining principal component analysis (PCA) with multiple linear regression. Its basic assumption is that the pollutant concentration at the receptor equals the sum of contributions from various pollution sources [25]. The calculation procedure is as follows: potential pollution sources affecting PTEs are first identified using PCA. The extracted principal components, representing potential sources, are then linearly regressed against PTE concentrations. The regression coefficients are then used to calculate the contribution rate of each pollution source to PTE enrichment [26]. This method does not require prior knowledge of the number and types of pollution sources; potential sources can be inferred merely based on pollutant information at the receptor site. With the advantages of simple operation and relatively objective and reliable results, APCS-MLR has been widely adopted in relevant studies [27,28]. The basic algorithm of APCS-MLR is expressed as follows:

(1) $$Z_{\mathrm{ik}}=\frac{X_{ik}-C_{i}}{\sigma_{i}}$$

(2) $$Z_{\mathrm{ik}}=\sum_{j=1}^{p}W_{ij}P_{jk}$$

(3) $${\left(APCS\right)}_{jk}=P_{j0}-P_{jk}$$

(4) $$X_{ik}=A_{i0}+\sum_{j=1}^{p}A_{ij}{\left(APCS\right)}_{jk}$$

In the above equations, *X_ik_* represents the concentration of element *i* at sampling site *k*; *C_i_* is the mean concentration of element *i*; *σ_i_* is the standard deviation; *Z_ik_* is the standardized matrix; *j* denotes the number of factors; *W_ij_* is the factor loading matrix; *P_jk_* is the factor score matrix, indicating the score of factor *j* at site *k*; *P_j0_* is the factor score at the zero-pollution point, where the concentrations of all PTEs are zero, and its introduction aims to calculate the absolute principal component score (*APCS*)*_jk_*; *A_i0_* is the intercept term in the regression equation, and *A_ij_* is the linear regression coefficient of the *j*-th factor for element *i*. Finally, the main pollution sources are identified based on *W_ij_*, and the contribution ratios of different pollution sources are calculated using (*APCS*)*_jk_* and *A_ij_*.

#### 2.3.3. Machine Learning Methods

Three machine learning models, namely Random Forest (RF), Extreme Gradient Boosting (XGBoost), and Light Gradient Boosting Machine (LightGBM), were adopted to analyze the driving factors of soil cadmium. All three models exhibit excellent nonlinear fitting capability, can effectively capture the complex relationships between PTEs and multisource influencing factors, and can output feature importance, which makes them suitable for identifying key driving factors.

(1) RF

Random Forest (RF) is an ensemble decision tree model based on the Bagging strategy. It constructs multiple independent decision trees through bootstrap sampling and random feature selection, and finally outputs the regression result by averaging the predicted values of all trees. The model has strong anti-overfitting capability and high stability, and can directly provide the importance score of each factor. It has become a commonly used method for data-driven analysis in environmental research [29].

(2) XGBoost

XGBoost is a gradient boosting algorithm under the Boosting framework. It improves prediction accuracy by iteratively fitting residuals in a serial manner and introduces regularization terms to control model complexity. With high prediction accuracy and strong robustness, it can explore the nonlinear relationships between influencing factors and PTEs concentrations, and is well applicable to the modeling of high-dimensional environmental variables [30].

(3) LightGBM

LightGBM is an efficient optimized version of the traditional gradient boosting decision tree. It adopts Gradient-based One-Side Sampling and Exclusive Feature Bundling strategies to improve computational efficiency, and uses a leaf-wise growth strategy to enhance fitting performance. The model features fast computation, low memory consumption, and good adaptability to large datasets. It improves modeling efficiency while maintaining prediction accuracy, and is suitable for analyzing the driving mechanisms of multi-factor interactions [31].

#### 2.3.4. Model Evaluation

In the machine learning-based analysis, all samples were randomly split into a training set (70%) and a test set (30%). The training set was used for model training, while the test set was used to independently evaluate the generalization and prediction performance of the model. To avoid overfitting and improve model stability, 10-fold cross-validation was performed only within the training set. The training set was evenly divided into ten subsets; in each iteration, nine subsets were used as the sub-training set, and one as the validation set, and the procedure was repeated ten times to complete model optimization. Three statistical indicators, including mean absolute error (MAE), root mean square error (RMSE), and coefficient of determination (R^2^), were employed to quantitatively evaluate the model prediction accuracy. In general, smaller values of MAE and RMSE and an R^2^ value closer to 1 indicate better model performance [32]. The used evaluation metrics are expressed by the following formulas, where *y_p_* denotes the predicted value, *y_o_* denotes the observed (true) value, *y^’^_p_* denotes the mean of the predicted values, and *N* denotes the total number of data points.

(5) $$MAE=\frac{\sum_{i=1}^{N}\left|y_{p}-y_{o}\right|}{N}$$

(6) $$RMSE=\sqrt{\frac{1}{N}\sum_{i=1}^{N}{\left(y_{p}-y_{o}\right)}^{2}}$$

(7) $$R^{2}=1-\frac{\sum_{i=1}^{N}{\left(y_{o}-y_{p}\right)}^{2}}{\sum_{i=1}^{N}{\left(y_{o}-{y^{,}}_{p}\right)}^{2}}$$

## 3. Results and Discussion

### 3.1. Basic Characteristics of PTE Concentrations

#### 3.1.1. Descriptive Statistical Analysis

As shown in Table 1, compared with the soil background values of Hunan Province, only Cd, Cr, and Ni exhibited enrichment characteristics in the study area, while the remaining PTEs showed no significant accumulation trend. The average contents of As, Cu, and Zn were 11.97 mg/kg, 24.15 mg/kg, and 76.77 mg/kg, respectively, all lower than the corresponding soil background values of Hunan Province. The average Pb content was 29.56 mg/kg, which was basically consistent with the regional background value of 29.70 mg/kg. The average concentrations of Cr and Ni were 81.01 mg/kg and 49.25 mg/kg, 1.13 and 1.54 times the background values, indicating slight accumulation to varying degrees. The average Cd content reached 0.43 mg/kg, 3.41 times the soil background value of Hunan Province. In addition, the average concentration of Cd was higher than the corresponding risk screening value of China (0.4 mg/kg), implying existing environmental risk. Cd was identified as the heavy metal with the most prominent enrichment degree in the study area.

#### 3.1.2. Spatial Distribution Patterns of PTEs in Soil

**Figure 2:**
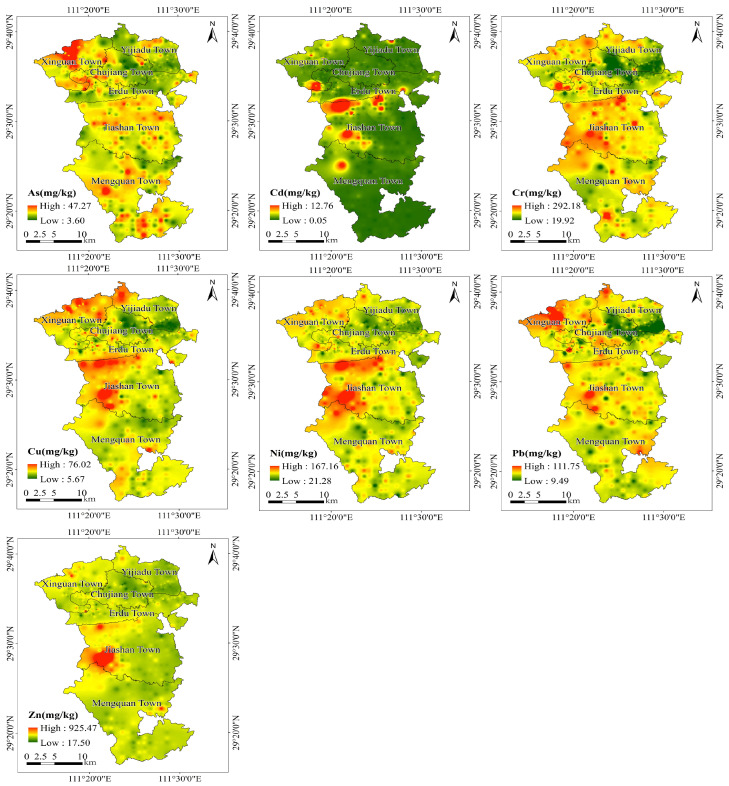
Spatial distribution pattern of PTEs in soil.

Inverse distance weighting (IDW) spatial interpolation was applied to systematically analyze the spatial distribution patterns of seven PTEs in the study area (Figure 2). The results indicated that all PTEs exhibited significant spatial differentiation characteristics: High arsenic (As) concentrations were mainly concentrated in Xinguan Town in the northwestern part of the study area, with scattered local high-value patches also observed in Mengquan Town and Jiashan Town. Overall, As levels were generally low across the entire study area, and the extent of areas exceeding the regional soil background value was limited. Cadmium (Cd) showed the most prominent spatial enrichment characteristics. The core high-concentration zone was located in the central part of the study area, concentrated in the northern and western parts of Jiashan Town. Meanwhile, certain Cd high-value areas also developed in some local zones in the northern part of the study area, presenting an obvious spatial agglomeration effect. Chromium (Cr), copper (Cu), nickel (Ni), lead (Pb), and zinc (Zn) showed strong spatial distribution similarity. Their high-concentration zones were mainly contiguous in the northern and central parts of the study area, with notable spatial continuity; meanwhile, some scattered high-value patches were also observed in local areas of the southern part.

### 3.2. Results of Multivariate Statistical Analysis

#### 3.2.1. Correlation Analysis (CA)

**Figure 3:**
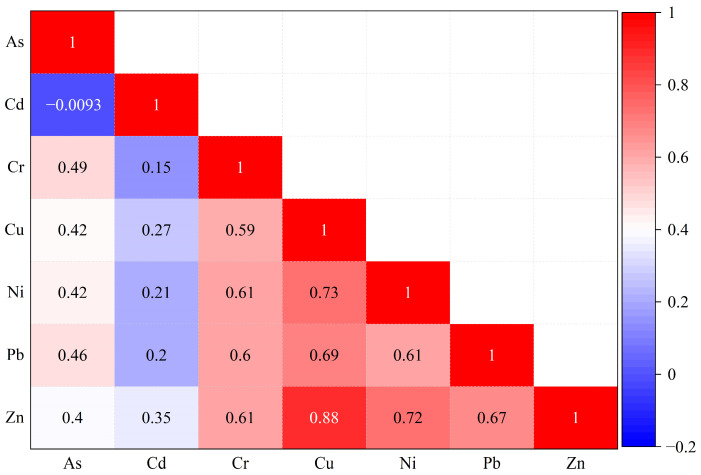
The correlation matrix of PTEs.

The correlation matrix helps explore relationships among variables by revealing the overall coherence of the dataset (Figure 3). Significant correlations were observed among most PTEs, indicating potential common pollution sources [35]. Cd had no significant correlation with As, but was moderately correlated with other PTEs, with correlation coefficients ranging from 0.15 to 0.35. In contrast, As was correlated with all PTEs except Cd, with correlation coefficients higher than 0.4, suggesting similar source contributions among these elements. Cr, Cu, Ni, Pb, and Zn exhibited strong intercorrelations, implying highly homologous pollution sources, with their correlation coefficients generally above 0.6. Notably, the correlation coefficient between Cu and Zn reached 0.88, revealing an extremely high similarity in their pollution sources.

#### 3.2.2. Principal Component Analysis (PCA)

PCA was applied to elucidate the origins of PTEs by reducing the original dataset to several dominant factors [10]. The data were assessed by KMO and Bartlett’s test. Results showed that the KMO value was 0.843 (>0.5) and Bartlett’s test was significant (<0.05), which indicated that the data was suitable for PCA.

Principal component analysis (PCA) was performed on seven PTEs, and a total of three principal components were extracted. These three principal components collectively explained 80.675% of the total data variance. The variance contribution rate of the first principal component was 35.554%, that of the second principal component was 25.574%, and that of the third principal component was 19.546%. As shown in Table 2, three principal components were identified in total. The first principal component had the highest loadings on Cu, Ni, and Zn; the second principal component exhibited relatively high loadings on As, Cr, and Pb; the third principal component showed the maximum loading on Cd, and also exerted a certain influence on Cr and Ni. Cluster analysis was also conducted (Figure S2). Results showed that there were three clusters—(1) Cr–Cu–Ni–Zn; (2) As, Pb; and (3) Cd—which was generally consistent with the PCA results.

### 3.3. Source Apportionment of PTEs in Soil

**Figure 4:**
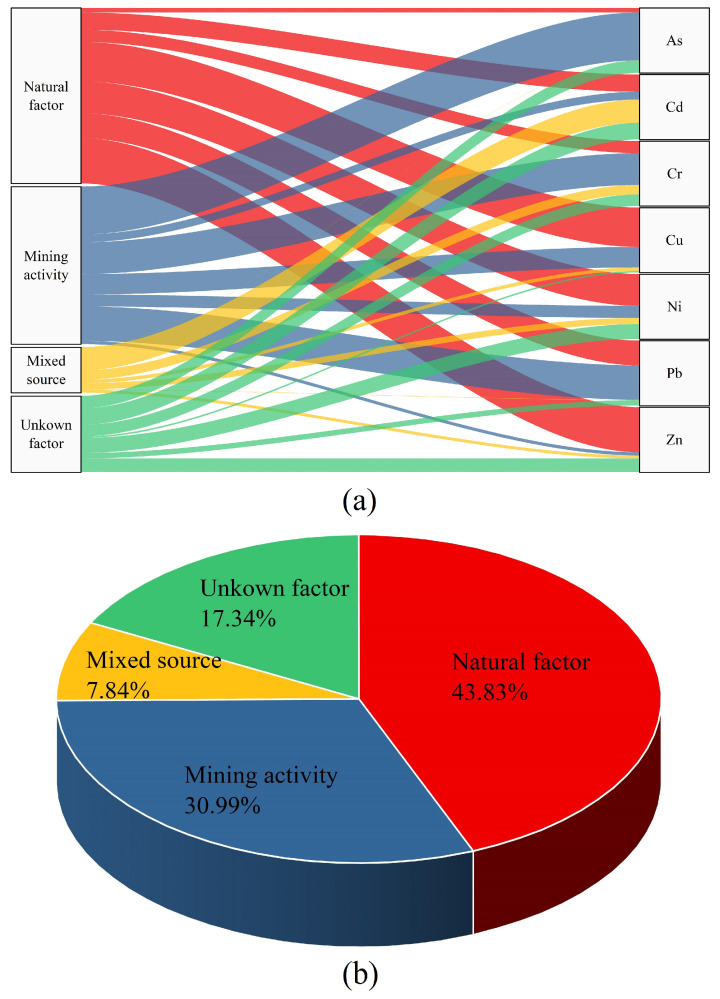
Pollution source contribution obtained by APCS-MLR. (**a**): Sankey diagram of pollution source contributions; (**b**) pie chart of average contribution rates of pollution sources.

Quantitative source apportionment of PTE pollution sources in the study area was conducted based on the APCS-MLR model (Figure 4). Source 1 contributed the largest proportion to Cu, Ni, and Zn, with contribution rates all exceeding 50%; particularly, its contribution rate to Zn reached as high as 69%. In addition, Source 1 also showed moderate contributions to Cd and Pb, with contribution proportions of 26.68% and 38.22%, respectively. In combination with the average concentrations of PTEs in the study area, the contents of most PTEs were close to the background values of Hunan Province, with no obvious accumulation. The natural background levels of PTEs in soil are primarily inherited from parent materials of soil formation, and elements released during rock weathering enter the soil, serving as an important potential source of PTEs in soil [36]. In this study, Source 1 presented a certain contribution to almost all PTEs, and the concentrations of most PTEs were close to the local background values. Therefore, Source 1 was identified as the natural source, with an average contribution rate of 43.83%.

**Figure 5:**
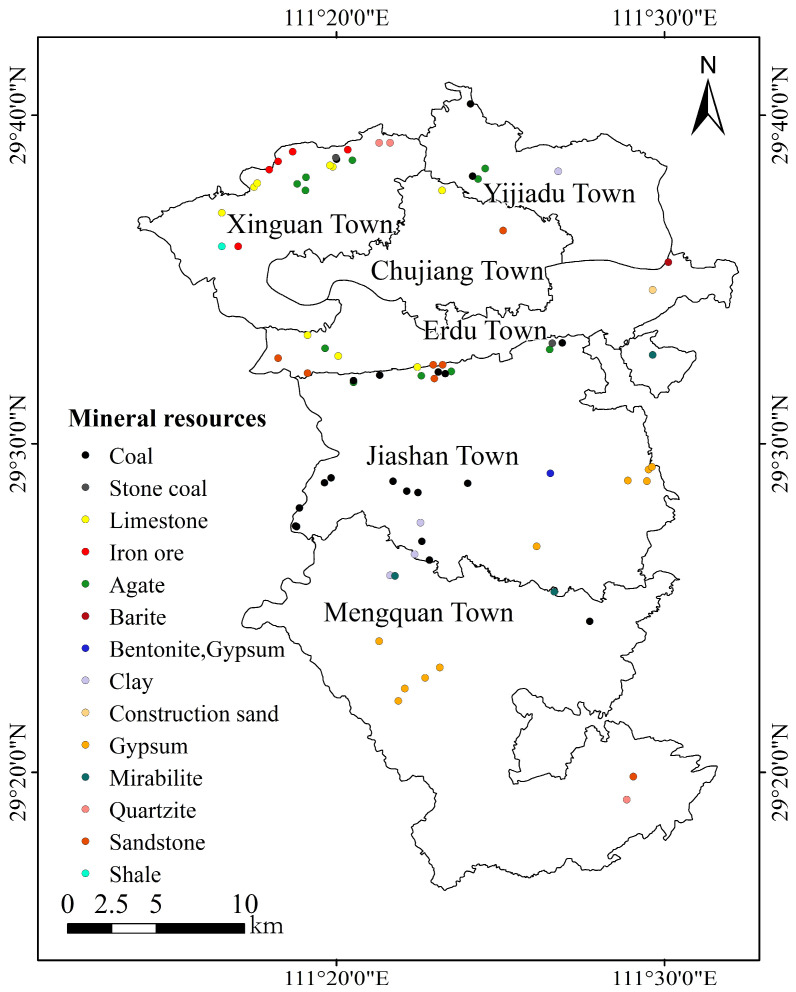
Spatial distribution of mineral resources in the study area.

Source 2 exhibited the highest contribution proportion to As at 73.72%, and also made considerable contributions to Cr (49.05%), Cu (31.41%), and Pb (52.87%). According to the spatial distribution characteristics of As, its high-value areas were mainly concentrated in Xinguan Town in the northern part of the study area. Based on the mineral distribution map of the study area (Figure 5), mineral resources are concentrated in the northern part of Xinguan Town, with limestone, iron ore, and stone coal as the dominant types. According to this spatial coupling relationship, it can be inferred that the high-value zones of PTEs in this area are likely closely associated with local mining activities. Previous studies have indicated that the study area is affected by various mining activities, including stone coal mining, hematite mining, and limestone mining [37]. Stone coal mining can elevate heavy metal concentrations in surrounding soils. A previous investigation on soils around stone coal mines in the lower reaches of the Zijiang River, Hunan Province, revealed that the contents of As, Cd, Cr, Cu, Ni, Pb, Zn, and Hg in the surrounding soils all exceeded the soil background values of Hunan Province. Areas with high PTE concentrations were mainly distributed in the central part of the study area, close to the concentrated distribution zone of stone coal mines [38]. After hematite mining, potentially toxic elements in mine soils may pose severe ecological risks to the surrounding areas. For instance, a study focusing on a typical abandoned open-pit iron mine along the Yangtze River found that combined pollution of potentially toxic elements posed significant carcinogenic and non-carcinogenic health risks to children [39]. Meanwhile, limestone is rich in carbonate minerals and tends to cause the enrichment of PTEs [40]. In summary, Source 2 was identified as mining activities, with an average contribution rate of 30.99%.

Source 3 contributed predominantly to Cd with a contribution rate of 35.65%, while its contributions to other PTEs were relatively low. Shimen County is one of the major citrus-producing areas in Hunan Province and even nationwide. The overlay analysis of Cd spatial distribution and land use types showed that several high-value areas of Cd were distributed in orchard zones. These regions feature intensive agricultural activities, which exert a remarkable influence on the accumulation of PTEs and serve as an important anthropogenic source of PTE pollution [41,42]. Pesticides and chemical fertilizers are inevitably applied in orchards. Numerous studies have confirmed that the application of agrochemicals and fertilizers is a critical pathway for PTE accumulation in soil [43,44]. In particular, phosphate fertilizers generally contain high concentrations of Cd because cadmium naturally exists in phosphate rock raw materials; long-term application can result in severe Cd accumulation in soil [45]. Furthermore, Cd hotspots were highly consistent with the distribution of coal mines (Figure 5), indicating that coal mining activities contribute significantly to Cd accumulation. Previous studies have also confirmed that coal mining and processing can promote the release and enrichment of Cd [46]. Accordingly, Source 3 was identified as a mixed source dominated by coal mining and agricultural activities, with an average contribution rate of 7.84% to PTE accumulation in the study area.

In addition, the intercept in the APCS-MLR model represents unidentified pollution sources [11,47]. This indicates that certain pollution sources remain unrecognized in the model. Such unidentified sources also exert considerable influence on PTEs, including As, Cd, Cr, Ni, and Zn, and play an important role in the accumulation of these elements, with an average contribution rate of 17.34%. Further research combining more analytical methods is required to achieve more precise apportionment of these unknown pollution sources in subsequent studies.

### 3.4. Identifying Driving Factors of Cadmium Using Machine Learning

The APCS-MLR results clarified source contributions, but the spatial distribution of Cd was further shaped by multi-factor interactions. Machine learning was therefore employed to unravel dominant drivers of Cd enrichment. Three tree-based models, namely Random Forest (RF), Extreme Gradient Boosting (XGBoost), and Light Gradient Boosting Machine (LightGBM), were adopted to simulate PTE concentrations in the study area. The comparison results of model performance are presented in Table 3. All three models showed a moderate overall prediction performance. Among them, the Random Forest (RF) model achieved the best performance, with a coefficient of determination (R^2^) of 0.28 on the validation set, while the MAE and RMSE were 0.17 and 0.25, respectively. The feature importance derived from a single training–test split is susceptible to sample randomness and may lead to biased results. Therefore, instead of adopting the outcome of a single data split, this study applied 10-fold cross-validation to repeatedly calculate the importance of each driving factor and took the average value. The results showed that the ranking of factor importance across all rounds of cross-validation was highly consistent, indicating that the identified key driving factors were not affected by random sample partitioning. The ranking results exhibited good robustness and reliability.

**Figure 6:**
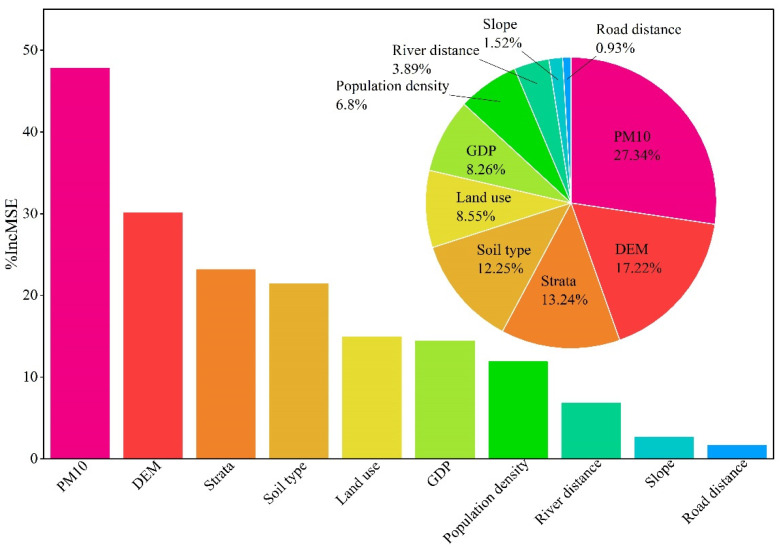
Driving factor importance ranking and relative contribution.

The bar chart (Figure 6) presents the %IncMSE value of each factor derived from the Random Forest model, which reflects its contribution to model prediction accuracy [48]. The pie chart (Figure 6) illustrates the proportion of normalized relative importance of each factor. The results showed that among the ten influencing factors included in the model, PM10 (27.34%), DEM (17.22%), Strata (13.24%), and Soil type (12.25%) were the dominant factors affecting Cd content, and the cumulative relative importance of these four factors reached approximately 70.05%. This conclusion is generally consistent with the findings of previous studies based on the geographical detector model [49]. To further clarify the relative contributions of natural and anthropogenic regimes, driving factors were divided into natural and anthropogenic groups, and their relative importance values were summed. Collectively, natural factors (strata, soil type, DEM, and slope) accounted for 44.23% of the total relative importance, whereas anthropogenic factors (PM10, land use, GDP, population density, river distance, and road distance) made up the remaining 55.77%. This contribution structure was highly consistent with the APCS-MLR source apportionment results. The consistent outcomes confirm that Cd enrichment in the study area was co-dominated by natural factors and anthropogenic activities.

Atmospheric deposition is an important pathway for the exogenous input of PTEs into soil, and is closely associated with intensive human activities such as mining–smelting emissions and traffic exhaust [50]. Mining and smelting activities, fossil fuel combustion, and traffic emissions release large quantities of PTE-laden atmospheric particles. Due to their large specific surface area, these particles readily adsorb and accumulate various PTEs, and eventually diffuse and settle onto the land surface via wind transport and precipitation leaching [51,52].

Topography and landforms are key natural factors driving the spatial differentiation of PTEs in soil. Their mechanism mainly lies in regulating the migration processes of PTEs and reshaping their geographical distribution patterns [7]. Driven by hydrodynamic conditions, PTEs tend to migrate and accumulate in low-lying areas along with surface runoff, forming a distribution pattern characterized by loss at high terrain and accumulation at low terrain [53]. Meanwhile, topography indirectly affects the input and accumulation of PTEs through multiple coupling effects. For example, atmospheric dust from industrial sources is easily blocked by terrain, resulting in a significant reduction in deposition flux at high altitudes or in enclosed terrain [51].

The lithological composition and elemental geochemical background vary among different strata in the study area. The background contents of PTEs in soil are mainly inherited from soil-forming parent materials, and PTE concentrations differ significantly across various parent materials [54]. During weathering and pedogenesis of different lithostratigraphic units, differences in mineral composition and elemental concentrations are directly transferred to overlying soils, resulting in distinct variations in the initial contents and enrichment characteristics of PTEs in soils derived from different strata [55]. Therefore, stratigraphic lithology serves as a core natural driving factor controlling the spatial distribution pattern of PTEs in soil, and lithological differences constitute an important cause of the spatial heterogeneity of PTE concentrations in the study area. In this study, the Permian strata exhibited the highest average Cd concentration at 1.17 mg/kg, followed by the Triassic strata with an average Cd content of 0.66 mg/kg (Table S2, Figure S3). Previous studies have reported relatively high Cd geochemical background values in Permian and Triassic rocks [56]. Earlier research supports the present findings, confirming that Permian carbonate rocks possess inherently high Cd geochemical background levels. Under intense chemical weathering, massive formation of Fe–Mn oxides and the transformation of dissolved organic matter toward microbial-derived components produce a synergistic effect, which collectively drives substantial Cd enrichment in soils [57].

There are differences in physicochemical properties among various soil types, including soil texture, pH value, organic matter content, clay content, and cation exchange capacity (CEC). These properties collectively control the adsorption, desorption, migration, and transformation processes of PTEs in soil [58]. It has been proven that pH strongly influences Cd migration [59]. Organic matter modulates Cd adsorption via adjusting soil pH, and CEC plays an important role in this process as well [60,61]. Accordingly, the accumulation characteristics and spatial differentiation of PTEs vary markedly across different soil types. In this study, limestone soil had the highest average Cd concentration of 1.17 mg/kg, while paddy soil showed an average Cd content of 0.43 mg/kg (Table S3, Figure S4). Limestone belongs to carbonate rocks. The pronounced Cd enrichment in soils derived from carbonate rock weathering is largely related to the intensive leaching of calcium ions during weathering, resulting in a generally high geochemical background of Cd in carbonate rock areas [62]. In addition, most soils in southern China are acidic, and the pH value of paddy soils exerts a significant influence on Cd enrichment [63].

Land use type, GDP, and population density also exert important impacts on the accumulation of soil Cd in the study area. Agricultural inputs and the intensity of anthropogenic disturbance vary under different land use patterns [64]. In this study, the average Cd concentration in orchard land was 0.47 mg/kg, while both paddy field and dry land had an average Cd content of 0.43 mg/kg; all were significantly higher than that of forest land at 0.28 mg/kg (Table S4, Figure S5). Orchard land, paddy field and dry land are more intensely affected by agricultural activities such as tillage, fertilization, and pesticide application, leading to a higher degree of soil Cd enrichment. GDP and population density can effectively characterize the intensity of regional human activities. Generally, higher GDP and greater population density correspond to stronger industrialization, agricultural intensification, and traffic intensity, which in turn increase the exogenous input and accumulation risk of PTEs [65,66].

### 3.5. Limitations and Future Perspectives

This study first systematically traced the potential sources of PTE pollution in the study area using multivariate statistical analysis and the APCS-MLR receptor model. Further focusing on the spatial differentiation characteristics of Cd, machine learning models were applied to reveal its nonlinear driving mechanisms in depth. Although this study has basically clarified the source composition and dominant driving laws of PTE pollution in the study area, certain limitations still exist due to constraints of data availability and research scale. First, the overall coefficient of determination (R^2^) of the machine learning models was relatively low, ranging from 0.21 to 0.28. This is attributable to the fact that PTEs are affected by the combined influence of geological background, mining activities, and agricultural activities, accompanied by strong spatial heterogeneity. The spatial enrichment of PTEs is co-regulated by multiple natural and anthropogenic factors. However, restricted by field monitoring conditions and data accessibility, it was difficult for this study to cover all potential influencing indicators comprehensively [20]. In addition, the spatial scale of this study is relatively small, making it difficult to obtain refined driving data related to anthropogenic activities. Some factors associated with agricultural production could not be included in the model analysis due to data gaps, which, to a certain extent, affected the fitting performance of machine learning models and the interpretation accuracy of driving mechanisms [17,32]. It should be noted that the core objective of this study was not to construct a high-precision prediction model, but to identify the relative importance of driving factors. The stable factor ranking obtained under cross-validation indicates that the model is reliable in identifying key driving factors. Second, the spatial resolution matching of multi-source data remains insufficient. The multi-source environmental and socioeconomic data adopted in this study, including atmospheric deposition, population density, and GDP, were extracted by clipping national-scale datasets. Although these data can generally characterize the spatial differentiation of relevant indicators in the study area, their refinement is limited when applied to small-scale regional research. The inadequate spatial resolution matching interferes to some extent with the stability of source apportionment results and the simulation accuracy of machine learning models [49]. Third, soil physicochemical indices were not adopted in the driving factor analysis. These properties are key factors governing Cd migration and adsorption. Restricted by data availability, we did not carry out relevant analysis, leaving room for further exploration of the influencing mechanisms in future research.

In view of the above deficiencies, future research will further improve the data foundation for source apportionment and driving mechanism identification of PTEs. More comprehensive and refined datasets of natural and anthropogenic driving factors will be collected as far as possible, and the spatial resolution of various grid and statistical data will be unified to ensure the consistency of multi-source data matching. In follow-up research, we will include soil physicochemical data to improve analytical depth and further clarify the mechanisms of Cd migration, adsorption and enrichment in soil. An improved data foundation can, on the one hand, provide more solid support for the APCS-MLR receptor model and improve the accuracy of pollution source identification and contribution quantification. On the other hand, it can effectively optimize the fitting performance of machine learning models and the interpretation accuracy of nonlinear driving mechanisms. This will further enhance the scientificity and reliability of the results regarding pollution source tracing and driving mechanism research of PTEs and provide more accurate theoretical basis and data support for the targeted prevention, control, and comprehensive management of regional PTE pollution.

## 4. Conclusions

This study investigated surface soils in southern Shimen County, coupling multivariate statistics with the APCS-MLR receptor model for quantitative source apportionment of PTEs, and further applying machine learning to systematically reveal the spatial enrichment patterns and dominant driving mechanisms of soil Cd. PTE concentration statistics indicated that Cd contamination was the most prominent, with an average content that was 3.41 times the soil background value of Hunan Province, while other PTEs remained close to regional background levels with no significant anthropogenic enrichment. The APCS-MLR model identified three typical pollution sources. Natural sources, as the primary contributor, exerted a widespread influence on most PTEs, with an average contribution of 43.83%; mining activities dominated the enrichment of As, Cr, and Pb, contributing 30.99% on average; the mixed source of coal mining and agricultural activities was the key anthropogenic trigger for Cd accumulation, contributing 35.65% to Cd specifically and 7.84% overall. Unresolved mixed sources accounted for 17.34%. Driving factor ranking revealed that atmospheric deposition (PM10), topography, strata, and soil type were the core factors governing Cd spatial differentiation, collectively accounting for 70.05% of relative importance. Among the selected drivers, natural factors contributed 44.23%, and anthropogenic factors 55.77%, indicating that Cd enrichment was jointly governed by natural background and exogenous anthropogenic inputs. Overall, by integrating receptor-based source apportionment and machine learning, this study clarified the potential sources of PTEs in soil and the key controls on Cd enrichment in southern Shimen County, elucidating the synergistic effects of geological background, mining exploitation, and agricultural activities. The findings provide scientific support and theoretical reference for source control, zoned management, ecological risk early warning, and territorial ecological restoration in the study area.

## Figures and Tables

**Table 1:** Descriptive statistics of PTE concentrations (mg/kg).

PTEs	Min	Max	Mean	Median	SD	CV (%)	ABVs	SPRSVs
As	3.46	49.44	11.97	11.04	5.13	42.83	15.7	30
Cd	0.04	13.34	0.43	0.28	0.80	187.20	0.126	0.4
Cr	18.85	306.25	81.01	81.45	22.93	28.30	71.4	250
Cu	5.35	76.32	24.15	24.00	7.32	30.29	27.3	150
Ni	19.40	167.84	49.25	49.05	12.22	24.80	31.9	70
Pb	8.94	116.09	29.56	29.77	7.66	25.93	29.7	100
Zn	16.34	930.60	76.77	73.88	39.61	51.60	94.4	200

Abbreviations: SD, standard deviations; CV, coefficient of variation; ABVs: Average background values in Hunan province [33]; SPRSVs: Soil pollution risk screening values of China (GB15618-2018) [34].

**Table 2:** Rotated component matrix from PCA.

PTEs	PC1	PC2	PC3
As	0.116	**0.892**	−0.015
Cd	0.210	−0.053	**0.913**
Cr	0.281	**0.596**	0.528
Cu	**0.806**	0.367	0.200
Ni	**0.758**	0.271	0.426
Pb	0.579	**0.651**	−0.044
Zn	**0.890**	0.062	0.176
Explained variance (%)	35.554	25.574	19.546
Cumulative variance (%)	35.554	61.128	80.673

Bold text represents the highest loading value of each heavy metal for each principal component.

**Table 3:** Prediction accuracy among different machine learning models.

Models	MAE	RMSE	R^2^
RF	0.17	0.25	0.28
XGBoost	0.18	0.27	0.21
LightGBM	0.18	0.27	0.22

## Data Availability

The data that support the findings of this study are available upon request from the corresponding author.

## Supplementary Materials

The following supporting information can be downloaded at: https://www.mdpi.com/article/10.3390/toxics14060525/s1, Table S1: Data description and sources; Figure S1: Potential influence factors of PTEs; Figure S2: Cluster analysis of PTEs; Table S2: Descriptive statistics of Cd concentrations of different strata (mg/kg); Figure S3: Boxplots of Cd concentrations in different strata; Table S3: Descriptive statistics of Cd concentrations of different soil types (mg/kg); Figure S4: Boxplots of Cd concentrations of different soil types; Table S4: Descriptive statistics of Cd concentrations of different land use types (mg/kg); Figure S5: Boxplots of Cd concentrations of different land use types.
