# Supplementary material for: Unraveling the Origins and Drivers of Potentially Toxic Elements (PTEs): A Sequential Framework Integrating Receptor Model and Machine Learning
Source: Toxics. 2026 Jun 17;14(6):525. doi: 10.3390/toxics14060525 (PMC13308060; doi:10.3390/toxics14060525)
Supplement: Supplementary file 1 [file toxics-14-00525-s001.zip › toxics-4349766-supplementary.pdf]

Supplementary Information

Unraveling the Origins and Drivers of Potentially Toxic Elements (PTEs): A Sequential Framework Integrating Receptor Model and Machine Learning

Jingyun Wang [1,2,*], Xiaofeng Zhao [1], Jiufen Liu [1,*], Yunxian Yan [3], Wei Zhao [2], Chuanbo Xia [2], Jianye Zheng [2] and Jiwei Liu [2]

[1] Key Laboratory of Coupling Process and Effect of Natural Resources Elements, Beijing 100055, China

[2] Key Laboratory of Gold Mineralization Processes and Resource Utilization, MNR, Key Laboratory of Metallogenic Geological Process and Resource Utilization of Shandong Provincial, Shandong Institute of Geological Sciences, Jinan 250013, China

[3] Institute of Geographic Sciences and Natural Resources Research, Chinese Academy of Sciences, Beijing 100101, China

***** Correspondence: jyunwang@126.com (J.W.); 13863858360@163.com (J.L.)

Table S1. Data description and sources

Driving factor	Data format	Data source
Strata	Vector layer	China Geological Survey (https://www.cgs.gov.cn/)
Soil type	Vector layer	Resource and Environment Science Data Platform (https://www.resdc.cn/)
DEM	Raster layer	Resource and Environment Science Data Platform (https://www.resdc.cn/)
Slope	Raster layer	Slope data were derived from DEM data using ArcGIS 10.8
Land use	Vector layer	Tsinghua University (https://data-starcloud.pcl.ac.cn/)
GDP	Raster layer	Resource and Environment Science Data Platform (https://www.resdc.cn/)
Population density	Raster layer	Resource and Environment Science Data Platform (https://www.resdc.cn/)
PM10	Raster layer	Dr. Wei Jing and Prof. Li Zhanqing at the University of Maryland (https://zenodo.org/records/6449937)
Road distance	Raster layer	National Catalogue Service for Geographic Information of China (https://www.webmap.cn/commres.do?method=result25W)
River distance	Raster layer	National Catalogue Service for Geographic Information of China (https://www.webmap.cn/commres.do?method=result25W)

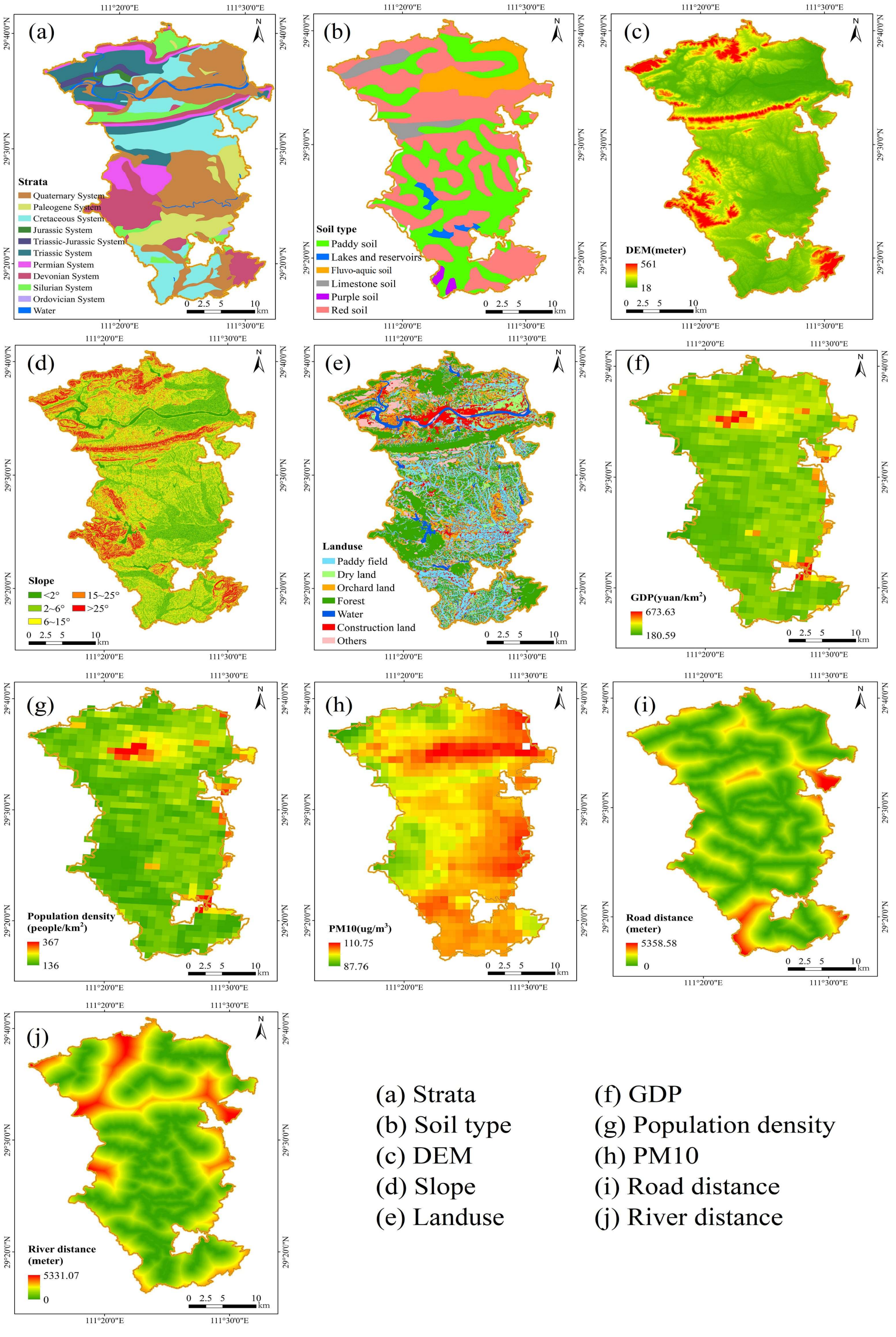

Figure S1. Potential influence factors of PTEs

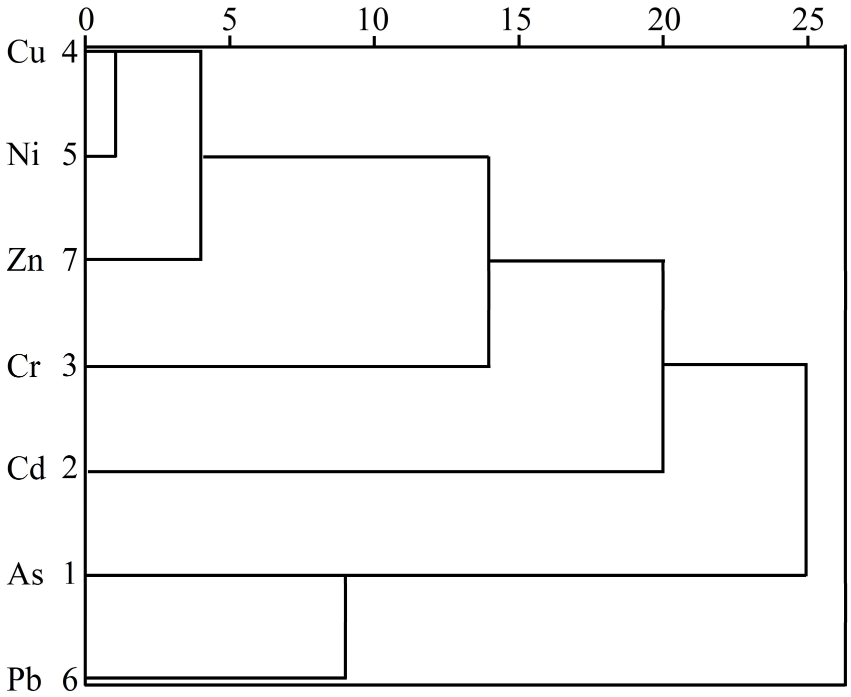

Figure S2. Cluster analysis of PTEs

Table S2. Descriptive statistics of Cd concentrations of different strata (mg/kg)

Strata	Min	Max	Mean	Median	SD	CV(%)
Quaternary system	0.07	1.85	0.32	0.28	0.21	63.94
Cretaceous system	0.04	4.10	0.40	0.25	0.60	150.71
Devonian system	0.16	2.31	0.57	0.20	0.86	152.02
Silurian system	0.13	0.94	0.27	0.21	0.18	68.36
Paleogene system	0.08	0.35	0.20	0.19	0.06	30.38
Permian system	0.13	13.34	1.17	0.64	2.26	193.09
Triassic system	0.08	12.06	0.66	0.35	1.28	194.30
Triassic–Jurassic system	0.10	0.10	0.42	0.34	0.23	54.37
Jurassic system	0.15	0.63	0.31	0.23	0.17	55.85

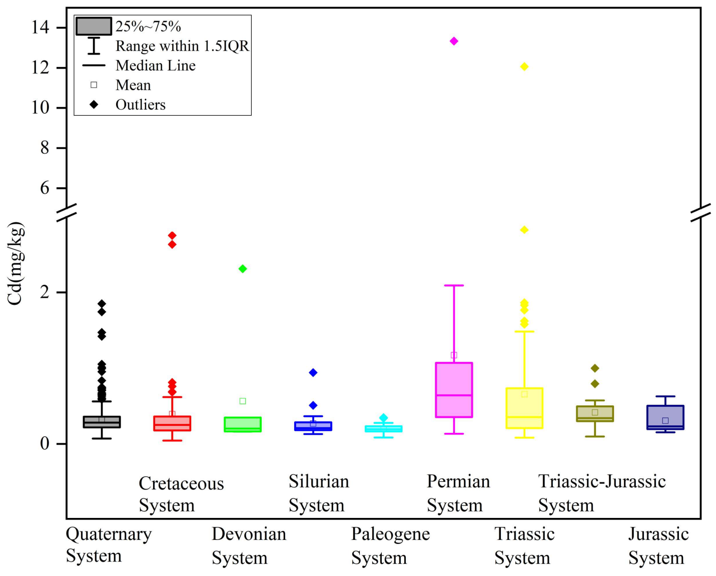

Figure S3. Boxplots of Cd concentrations in different strata

Table S3. Descriptive statistics of Cd concentrations of different soil types (mg/kg)

Soil type	Min	Max	Mean	Median	SD	CV(%)
Red soil	0.04	4.10	0.40	0.28	0.43	107.40
Paddy soil	0.08	13.34	0.43	0.25	0.99	229.22
Purple soil	0.09	0.21	0.16	0.15	0.05	28.54
Limestone soil	0.13	12.06	1.18	0.59	2.14	181.36
Fluvo-aquic soil	0.09	2.75	0.32	0.29	0.24	74.00

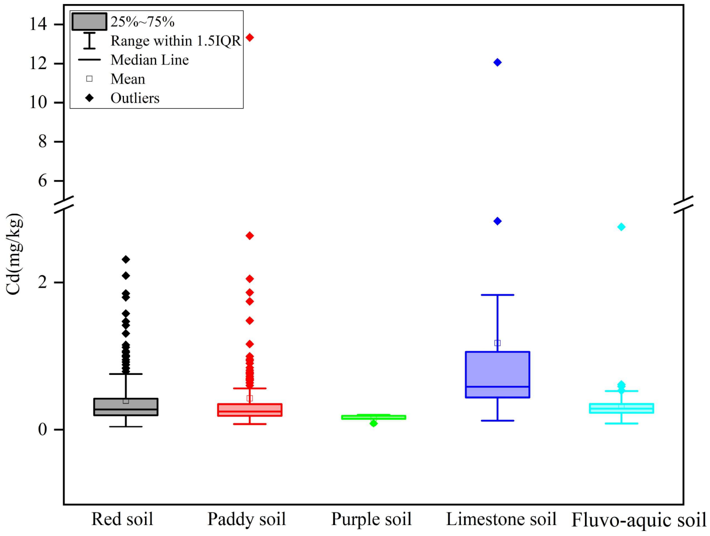

Figure S4. Boxplots of Cd concentrations of different soil types

Table S4. Descriptive statistics of Cd concentrations of different land use types (mg/kg)

Land use	Min	Max	Mean	Median	SD	CV(%)
Paddy field	0.04	4.38	0.43	0.27	0.53	122.90
Dry land	0.09	12.06	0.43	0.28	1.12	264.12
Forest	0.08	1.06	0.28	0.19	0.22	79.43
Orchard land	0.07	13.34	0.47	0.31	1.08	232.04
Construction land	0.12	0.89	0.32	0.30	0.16	51.19
Others	0.11	2.31	0.56	0.24	0.71	126.38

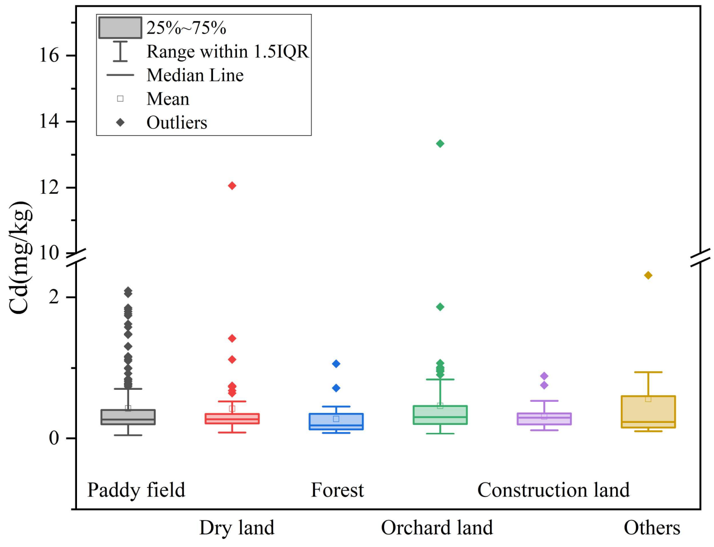

Figure S5. Boxplots of Cd concentrations of different land use types
